# D-OLIA: A Hybrid MPTCP Congestion Control Algorithm with Network Delay Estimation

**DOI:** 10.3390/s21175764

**Published:** 2021-08-27

**Authors:** Tabassum Lubna, Imtiaz Mahmud, Geon-Hwan Kim, You-Ze Cho

**Affiliations:** School of Electronic and Electrical Engineering, Kyungpook National University, Daegu 41566, Korea; lubna@knu.ac.kr (T.L.); imtiaz@knu.ac.kr (I.M.); kgh76@ee.knu.ac.kr (G.-H.K.)

**Keywords:** D-OLIA, MPTCP congestion control algorithm, network condition estimation, network delay estimation

## Abstract

With the recent evolution of mobile technology, modern devices equipped with multiple communication interfaces have become popular. The multipath transmission control protocol (MPTCP) has evolved to facilitate multiple communication interfaces through a single TCP connection for faster Internet access. MPTCP congestion control algorithms (MPTCP-CCAs) control data flow by fulfilling three design goals, i.e., ensuring improvement over single-path flows, ensuring fairness, and balancing congestion. Current MPTCP-CCAs cannot fulfill these design goals. For example, the opportunistic-linked increase algorithm (OLIA), a well-known MPTCP-CCA in load balancing, often results in low throughput because it cannot properly utilize the underlying network. In addition, the current Internet has a rapidly changing characteristic due to a large amount of short-lived traffic, making it difficult for MPTCP-CCAs to cope. An awareness of prevailing network delay conditions might help MPTCP-CCAs to utilize the network capacity fully. Therefore, we propose dynamic OLIA (D-OLIA), a hybrid MPTCP-CCA that enhances the performance of OLIA by integrating an awareness of the current network delay condition for deciding the congestion window (CWND) decrease factor. We estimate the current network delay condition, i.e., less-congested or congested, by observing the changes in the round-trip-time (RTT). Based on the estimated network delay condition, we decide the CWND decrease factor in real-time for reducing the CWND during packet loss events. We implemented D-OLIA in the Linux kernel and experimented using the Mininet emulator. The emulation results demonstrate that D-OLIA successfully estimates current network delay conditions and results in approximately a 20% increased throughput compared to the original OLIA. Compared to certain MPTCP-CCAs, it also yields a highly improved performance in terms of throughput, RTT, packet retransmissions, and fairness among the MPTCP sub-flows.

## 1. Introduction

Transmission Control Protocol (TCP) is a transport layer protocol widely used for its reliability and fairness in competing with other flows on the Internet for decades [1]. TCP aims at the proper utilization of the available bandwidth (BW) [2]. At present, due to the immense improvement in wireless communication technology, multi-homed mobile terminals equipped with multiple communication interfaces can access various wired/wireless networks simultaneously [3]. Therefore, to cope with the growing development of network capacity, TCP has extended from using a single-path to using multiple paths of TCP connection. Multipath TCP (MPTCP) is a significant modification of single-path TCP proposed by the Internet Engineering Task Force (IETF) working group. MPTCP allows for the devices to initiate multiple flows through multiple paths in a single TCP connection [4]. Thus, it provides a faster Internet experience and reliability [2].

TCP uses congestion control algorithms (CCA) to determine the amount of in-flight data to control data flow to ensure network stability and higher BW utilization. Similarly, for MPTCP, MPTCP-CCAs are used to utilize the underlying network properly. For developing a MPTCP-CCA, three primary design goals need to be fulfilled [5].

- Goal 1 (improve throughput): A multipath flow should perform at least as well as a single-path flow would on the best of the paths available to it. This goal ensures that there is an incentive for deploying multipaths.
- Goal 2 (not harm): A multipath flow should not take up any more capacity on any one of its paths than if it were a single-path flow using only that route. This goal guarantees that it will not unduly harm other flows.
- Goal 3 (balance congestion): a multipath flow should move as much traffic as possible off its most-congested paths, subject to meeting the first two goals.

By following these design goals, several researchers have proposed various MPTCP-CCAs. However, if we categorically consider the TCP-CCAs, there are mainly three variations of TCP-CCAs: loss-based, delay-based, and hybrid CCAs [6]. According to RFC 793 [7], Tahoe [8], Reno [9], and NewReno [10], binary increase congestion control (BIC) [11] and Cubic [12] are considered loss-based TCP-CCAs. Vegas [13], FAST [14], low latency congestion control (Lola) [15], and Timely [16] are known as delay-based TCP-CCAs. Vegas-reno (Veno) [17], Compound [18], Fusion [19], Illinois [20], bottleneck bandwidth and round trip time (BBR) [21], and performance-oriented congestion control (PCC) [22] are considered hybrid TCP-CCAs. In RFC 6824, MPTCP is considered a next-generation transport layer protocol [23]. We can divide MPTCP-CCAs into three main groups: (a) loss-based MPTCP-CCA, such as linked increased algorithm (LIA) [5], opportunistic linked increased algorithm (OLIA) [24], balanced linked adaptation (BALIA) [25], and dynamic LIA (D-LIA) [26]; (b) delay-based MPTCP-CCAs, such as weighted Vegas (wVegas) [27] and coupled multipath BBR (C-MPBBR) [26]; and (c) hybrid MPTCP-CCAs, such as the multipath compound (MCompound) [28]. However, a widely accepted MPTCP-CCA is still missing. Currently, a considerable amount of research has been focusing on fulfilling the demand for an efficient and standard MPTCP-CCA by satisfying all the design goals.

Due to long-distance work and live streaming demands, reliable and faster Internet has become crucial. Furthermore, given the high volume of short-lived traffic, network conditions change continuously. Several high-end research works have been going on to address this issue [29,30,31,32,33,34,35,36]. We firmly believe that considering current network conditions can play a vital role in ensuring high throughput and reducing delay over the Internet. In this regard, a high-performance MPTCP-CCA that estimates and considers the network’s current delay condition and reacts accordingly might be a better solution for the required high-speed and reliable Internet. 

In this paper, we propose a novel hybrid MPTCP-CCA named Dynamic OLIA (D-OLIA), which is a combination of the loss-based and delay-based congestion control approaches. By satisfying the three design goals, D-OLIA ensures high throughput with reduced delay. Furthermore, it performs improved load balancing by ensuring fairness among the MPTCP SFs. The key contributions of this paper are listed below.

1. D-OLIA estimates the current network condition by categorizing the network into two categories, i.e., a less-congested and a congested network.
2. D-OLIA adjusts the congestion window (CWND) decrease factor in ensuring high bandwidth utilization and low delay considering the current network condition.
3. Simultaneously, D-OLIA ensures that all the SFs equally and efficiently use the underlying network.
4. D-OLIA also tries to meet the three design goals of MPTCP by ensuring high throughput, fairness, and successful load balancing.
5. D-OLIA improves the performance of OLIA in terms of throughput and fairness among MPTCP SFs.
6. Furthermore, D-OLIA ensures comparatively better throughput, delay, and packet retransmissions than MPTCP-CCAs, such as LIA, BALIA, and D-LIA.

The organization of the rest of the paper is as follows: Section 2 summarizes the related works, Section 3 describes the motivation behind the proposal, Section 4 briefly describes the proposed D-OLIA CCA, and Section 5 evaluates the performance of D-OLIA in comparison with existing algorithms such as LIA, OLIA, BALIA, and D-LIA. Finally, Section 6 concludes the paper.

## 2. Related Works

This section briefly discusses some previously proposed major MPTCP-CCAs including their properties, advantages, and shortcomings. We can divide the existing MPTCP-CCAs into three main categories, i.e., loss-based, delay-based, and hybrid.

In loss-based MPTCP-CCAs, packet losses dictate the transmission rate. The MPTCP-CCAs consider packet loss to indicate congestion, i.e., whenever there is packet loss, they consider that the underlying bottleneck buffer is full. To respond to this congested network scenario, they reduce the CWND, which ultimately reduces the data flow so that the buffer becomes free. Following MPTCP’s primary design goals, several loss-based MPTCP-CCAs have been proposed such as LIA [5], OLIA [24], BALIA [25], and D-LIA [26].

Raiciu et al. proposed LIA to fulfill the design goals of MPTCP-CCAs [5]. LIA could successfully shift the traffic from a congested path to a less congested path while improving throughput and fairness. However, LIA fails to fully utilize the underlying network due to its insistence on fairness [37].

By analyzing LIA’s behavior, Khalili et al. identified that LIA forces a tradeoff between optimal congestion balancing and responsiveness. To eliminate this tradeoff and simultaneously provide these characteristics, they proposed OLIA [24]. However, OLIA also faces the issue of underutilizing the underlying network [37].

Although OLIA aimed at providing optimal load balancing and responsiveness simultaneously, Peng et al. reported that OLIA sometimes shows unresponsiveness to changes in the network conditions depending on various network scenarios. To mitigate this concern, they proposed a modified algorithm named BALIA [25]. However, the issue of underutilization of the underlying network persists in BALIA as well [37].

These previously proposed MPTCP-CCAs fail to utilize the underlying network properly, resulting in more inadequate network throughput [37]. Furthermore, these algorithms only focused on the CWND increase mechanism. To address this issue, we proposed D-LIA [26], a loss-based CCA that dynamically controls the CWND decrease mechanism. D-LIA adjusted the CWND decrease factor in packet losses by considering the interval between packet losses. D-LIA could achieve high throughput in comparison with LIA, OLIA, and BALIA. However, it showed a significant increase in packet losses because it showed slow responsiveness to network congestion.

By contrast, delay-based MPTCP-CCAs follow a proactive system. Delay-based CCAs use the delay as a congestion indicator and prevent queue buildup at the bottleneck buffer. Their main goal is to ensure minimum round-trip-time (RTT) and maximum throughput. Hence, they are best suited for low-latency applications. Popular delay-based MPTCP-CCAs include wVegas [27] and C-MPBBR [26].

Yu et al. proposed a delay-based MPTCP-CCA based on TCP Vegas and named it wVegas [27]. They considered packet queueing delay as the congestion signal and achieved fine-grained load balancing. Interestingly, rather than the three design goals of MPTCP, the authors of this work focused on fulfilling the “congestion equality principle”. As a result, it does not fulfill the design goals of MPTCP-CCAs.

To successfully fulfill the design goals of MPTCP-CCAs, we proposed a delay-based CCA for MPTCP based on single-path TCP BBR [21] and named it C-MPBBR [26]. In this work, we focused on fulfilling the design goals of MPTCP while ensuring high throughput, low delay, and improved fairness. However, the single-path TCP-CCA BBR [21] has specific critical issues that are still under development. Therefore, we expect a further improvement of C-MPBBR in the near future.

In the case of hybrid MPTCP-CCAs, we expect that a combined strategy of loss-based and delay-based CCAs might provide high throughput while fulfilling the design goals of MPTCP-CCAs. However, until now, no successful implementation of a hybrid approach for MPTCP-CCAs is present to the best of our knowledge. Phuong et al. proposed a hybrid MPTCP-CCA for high speed and long delay networks called MCompound [28]. It is a multipath implementation of the previously proposed Compound TCP [18] and achieves better throughput for high-speed and long-delay networks. However, it does not consider the design goals of MPTCP, i.e., it does not behave fairly with single-path TCP flows.

As a result, a standard MPTCP-CCA that fulfills all the MPTCP-CCA design goals is still missing. Therefore, we focus our research on filling this gap by presenting a novel hybrid MPTCP-CCA in this work.

## 3. Motivation

In this section, we briefly describe the motivation behind our proposed MPTCP-CCA. As discussed before, the current MPTCP-CCAs require further improvements for successfully handling the MPTCP sub-flows (SFs). We will discuss three issues that motivated us towards this proposal.

### 3.1. Issue I: Fairness among MPTCP SFs

**Figure 1:**
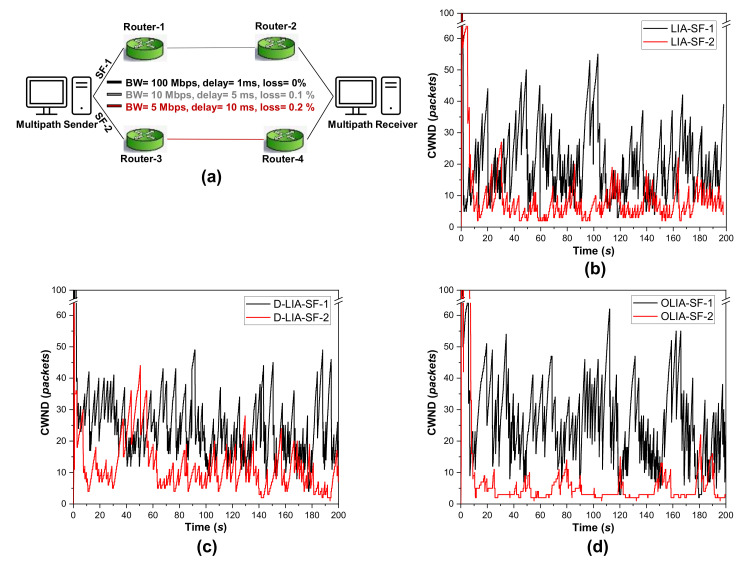
Simple experiment for observing the CWND changes for various MPTCP-CCAs: (**a**) the experimental scenario (**b**–**d**) when the applied MPTCP-CCA is LIA, D-LIA, and OLIA, respectively.

For describing this issue, we will conduct a simple experiment. Figure 1a shows the experimental scenario. There are two separate paths between a MPTCP sender and receiver. Thus, there are two SFs. For SF-1, the bottleneck BW is 10 Mbps, RTT is 14 ms, and the loss rate is 0.1%. The SF-2 has a bottleneck BW of 5 Mbps, an RTT of 24 ms, and a loss rate of 0.2%. We experimented for 200 s and tested LIA, D-LIA, and OLIA. Note that we configured the system such that both the paths were available to the sender and receiver from the beginning. However, following the connection formation process of MPTCP, SF-1 was started first and then SF-2 was started. Thus, SF-1 becomes the first flow and SF-2 becomes the second flow. Compared to the performance of LIA (Figure 1b) and D-LIA (Figure 1c), as we can observe from Figure 1d, SF-2 could never utilize the underlying network properly in OLIA. As a well-known MPTCP-CCA that ensures fairness with single-path flows, OLIA’s failure to use the underlying network for the later started flows makes OLIA significantly lag in utilizing all the available paths simultaneously. It results in low throughput than the single-path TCP flows intermittently. Therefore, we attempt to improve OLIA further by solving this problem through our proposal in Section 4.

### 3.2. Issue II: Network Delay Condition

When the bottleneck queue becomes full, the bottleneck discards all the packets that arrive later in the data transmission procedure. This process introduces a significant delay in transmitting the existing packets. However, when the queue is empty, the existing network capacity is wasted. Therefore, an intelligent method is necessary that can successfully estimate the network delay condition and feed the result to the CCA to control the data flow appropriately. However, in the current state of the Internet, it isn’t easy to measure precisely the current available queue size in the middleboxes from the end systems. The best approach is to apply a better estimation algorithm at the end systems. Therefore, we aim to design an efficient network delay condition estimator to improve the output of the MPTCP-CCAs. Primarily, we aim at successfully incorporating the output of the network delay estimator into OLIA so that OLIA can fulfill the three design goals of MPTCP-CCAs while better utilizing the bottleneck queue.

### 3.3. Issue III: Absence of Proper Hybrid MPTCP-CCA

As per the classification of TCP and MPTCP’s CCAs discussed in Section 1, MPTCP has only one hybrid CCA named mCompound, which was proposed especially for long-delay networks. Furthermore, mCompound does not consider the design goals of MPTCP-CCAs. We believe that to fulfill the demand for high-speed networks, a hybrid approach in combination with the delay-based and loss-based techniques would be a better candidate as an efficient MPTCP-CCA. This hybrid approach will enable the MPTCP-CCA to understand the underlying network better and feed a proper amount of data into it. Therefore, this work concentrates on a hybrid MPTCP-CCA that combines the delay-based and loss-based approaches and shows improved performance as a MPTCP-CCA.

## 4. Hybrid MPTCP-CCA: D-OLIA

As mentioned before, to cope with the expanding network traffic and dynamically changing network conditions, we believe that the MPTCP-CCA should be aware of the network situation before deciding the CWND. To accomplish this, we propose a new hybrid MPTCP-CCA, namely D-OLIA, which is a combination of delay-based and loss-based approaches.

In D-OLIA, we choose a hybrid approach for deciding the CWND size. During each loss event, we select the CWND decrease factor based on the network delay estimation.

### 4.1. Network Delay Condition Estimation

D-OLIA considers two network states of network delay conditions: the congested state and less-congested state. D-OLIA identifies these two network delay condition states by systematically evaluating the RTT changes.

RTT is considered a critical variable that helps to understand the bottleneck queue occupancy. When the bottleneck queue occupancy is low, the RTT becomes short as the packet can travel without waiting in the queue for a long time. When the bottleneck queue occupancy is high, the packets need to wait in the queue for a longer time, resulting in longer RTTs. Therefore, based on the changes in the RTT, the bottleneck queue occupancy can be estimated.

Now to relate it with the available RTT information, D-OLIA implements an RTT-probe mechanism. It sets an RTT-probe period of 0.5 s. During this RTT-probe period, D-OLIA measures the minimum RTT ($RTT_{\min}$) and maximum RTT ($RTT_{\max}$) as follows:

(1) $$RTT_{\min}=\min\left(RTT_{curr}\right.,\left.RTT_{\min}\right),$$

(2) $$RTT_{\max}=\max\left(RTT_{curr},\left.RTT_{\max}\right)\right.,$$

where $RTT_{curr}$ is the current RTT. After each RTT-probe period, D-OLIA finds the mid-point, $RTT_{mid}$, between the $RTT_{\min}$ and $RTT_{\max}$ as follows:

(3) $$RTT_{mid}=\left(RTT_{\min}+RTT_{\max}\right)/2$$

D-OLIA considers this $RTT_{mid}$ as a representation of the middle of the bottleneck buffer queue. Thus, if $RTT_{curr}>RTT_{mid}$ and $RTT_{curr}>RTT_{prev\_loss\_evnt}$, then D-OLIA considers that the network is now in a congested state and a less-congested state otherwise, where $RTT_{prev\_loss\_evnt}$ is the RTT of previous congestion. Note that D-OLIA updates the values of $RTT_{\min}$, $RTT_{\max}$, and $RTT_{mid}$ after each RTT-probe period of 0.5 s. Furthermore, D-OLIA considers whether it received more than three ACKs to ensure the consideration of enough samples for the measurements. Finally, $RTT_{prev\_loss\_evnt}$ is updated after each decrease of the CWND. Algorithm 1 summarizes the $RTT_{mid}$ calculation mechanism.


**Algorithm 1:***RTT_mid_*—Determination**Initialization:** *RTT_max_* = 0 *RTT_min_* = 9999 *num_of_received_ack* = 0 *system_update_time* = 0 **Upon reception of ACK:** **if** *RTT_curr_* < *RTT_min_* **then**   *RTT_min_* = *RTT_curr_* **end**
**if** **if** *RTT_curr_* > *RTT_max_* **then**   *RTT_max_* = *RTT_curr_* **end**
**if** *num_of_received_ack* = *num_of_received_ack* + 1 **if**
*num_of_received_ack* > 2 **and** *system_current_time* > *system_update_time* **then**   *RTT_mid_* = (*RTT_min_* + *RTT_max_*)/2   *RTT_max_* = *RTT_curr_*   *RTT_min_* = *RTT_curr_*   *num_of_received_ack* = 1   *system_update_time* = *system_current_time* + 0.5 **end if** **return** *RTT_mid_*


### 4.2. CWND Adjustment Mechanism

For the CWND selection mechanism, D-OLIA adopts the loss-based additive increase multiplicative decrease mechanism. For the CWND increase mechanism, D-OLIA simply implements OLIA’s CWND increase mechanism to incorporate OLIA’s improved fairness and load balancing process. Following OLIA’s CWND increase method, for a sub-flow *r*, D-OLIA increases the CWND ($w_{r}$) per ACK as shown below [24]:

(4) $$w_{r}=\left(\frac{w_{r}/rtt_{r}^{2}}{{\left(\sum_{p\in R}w_{p}/rtt_{p}\right)}^{2}}\right)+\left(\frac{\alpha_{r}}{w_{r}}\right)$$

For the CWND decrease mechanism for each packet loss event, based on the estimated network delay condition, D-OLIA implements either the standard OLIA’s CWND decrease mechanism, i.e., half of the CWND, or D-LIA’s [26] CWND dynamic decrease mechanism, i.e., dynamically adjust the CWND decrease factor based on the CWND of the current and previous loss events. 

In the case of a congested network, CWND needs to decrease sharply to let the queue become free quickly. To facilitate this, D-OLIA halves the CWND when the network condition is in a congested state. 

By contrast, the queue occupancy is less than half in the less-congested network state so that the network can process more packets. Therefore, D-OLIA implements D-LIA’s CWND decrease mechanism to facilitate a smooth decrease in the CWND. For a packet loss event, D-OLIA decreases the CWND (*w_r_*) by multiplying by a decreasing factor *β*, i.e., per loss event on a sub-flow *r*, shown as

(5) $$w_{r}=\max\left(\beta_{}*w_{r},1\right),$$

where β is bounded by $\beta_{\min}\leq \beta \leq \beta_{\max}$. D-OLIA defines β as follows:

(6) $$\beta =\left(0.25*\gamma \right)+\left(1-0.25\right)*\beta^{\prime },$$

where $\beta^{\prime }$ is the value of *β* at the previous loss event and *γ* is the ratio between the CWND at the previous loss event and the current loss event. *γ* is bounded by 0≤γ≤1 and defined by:

(7) $$\gamma =\min\left(w_{r}^{\prime }/w_{r},\;1\right),$$

where $w_{r}^{\prime }$ is the CWND at the previous loss event.

**Figure 2:**
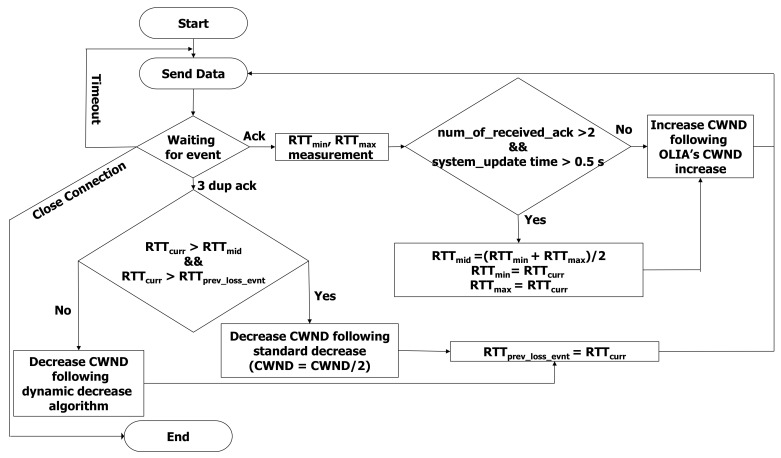
Flowchart of per-flow CWND adjustment process of the proposed D-OLIA algorithm.

Algorithm 2 summarizes the implementation of the dynamic decrease mechanism. The flowchart in Figure 2 summarizes the proposed hybrid MPTCP algorithm of D-OLIA.



**Algorithm 2:**
*CWND—Dynamic Decrease Mechanism*
**Initialization:** *β_prev_* = 0.5 *β_min_* = 0.5 *β_max_* = 0.9 *factor* = 0.25 *cwnd_prev-loss-evnt_* = 1 *RTT_prev-loss-evnt_* = 1 **Upon reception of 3 dup-ack:** *γ* = min (*cwnd_prev-loss-evnt_*/*cwnd*, 1) *β* = (*factor* * *γ*) + (1 − *factor*) * *β_prev_* **if** *β < β_min_* **then**   *β* = *β_min_* **end if** **if** *β* > *β_max_* **then**   *β* = *β_max_* **end if**
 *cwnd* = max (*β* * *cwnd*, 1) *cwnd_prev-loss-evnt_* = *cwnd* *β_prev_* = *β* *RTT_prev-loss-evnt_* = *RTT_curr_* **return** *cwnd*


### 4.3. Computational Complexity and Implementation of D-OLIA in the Linux Kernel

As D-OLIA adds simple logic to the existing OLIA, the added computational complexity of D-OLIA is minimum and can be expressed by O(1). We modified OLIA’s Linux Kernel code for the implementation of D-OLIA in the Linux kernel. Whenever the sender receives an ACK, D-OLIA updates the $RTT_{curr}$ and $RTT_{\max}$, and adjusts the CWND increase mechanism following the proposed algorithm. In the OLIA’s implementation in the Linux kernel, MPTCP calls the cong_avoid function upon reception of each ACK. Therefore, we modified this function to update the $RTT_{curr}$ and $RTT_{\max}$. In addition, $RTT_{mid}$ is calculated here upon fulfillment of the proposed condition.

On the contrary, the CWND decrease factor is decided only upon the reception of 3-duplicate ACKs. In OLIA’s implementation in the Linux kernel, MPTCP calls the ssthresh function for the 3-duplicate ACK events. Hence, we implemented the CWND decrease logic in this function. We also added the required parameters for calculating $RTT_{mid}$ and β in OLIA’s data structure.

## 5. Performance Evaluation

In this section, we first present the details of the experimental setup and then analyze the performance in terms of CWND, RTT, throughput, aggregate benefit, number of packet retransmissions, and fairness.

### 5.1. Experimental Setup

We evaluated and compared the performance via emulation experiments on a Linux network namespace-based Mininet emulator [38]. We used “ethtool” [39] and “NetEm” [40] for configuring the BW and RTT, respectively; “iperf3” [41] for transmitting the data and measuring the total throughput; “ifstat” [42] for measuring throughput per flow; and “tcpprobe” [43] for measuring the CWND. For the experiment, we used MPTCP v0.93.4 deployed in Linux Kernel v4.9.169.

**Figure 3:**
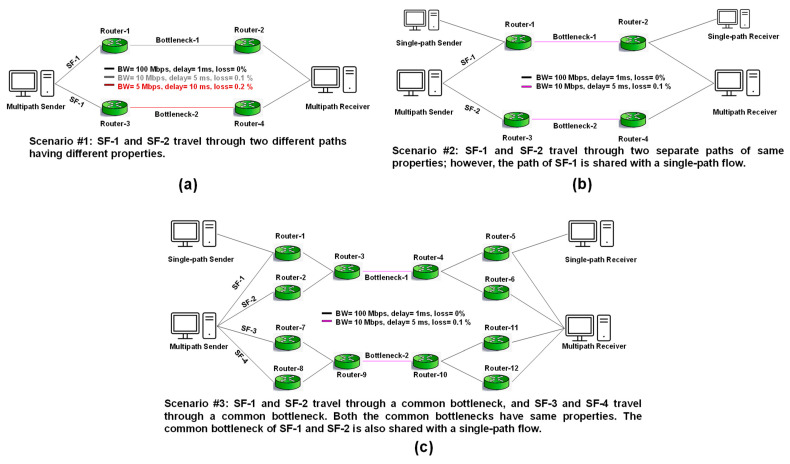
Illustration of the considered scenarios: (**a**) Scenario #1, (**b**) Scenario #2, and (**c**) Scenario #3.

We compared the performance of the proposed D-OLIA with some existing MPTCP-CCAs such as LIA, OLIA, BALIA, and D-LIA. We configured three emulation scenarios to observe and compare the performance of the considered MPTCP-CCAs, as shown in Figure 3. Single-path TCP flow having CUBIC as the CCA was used as the background traffic during the experiments unless specified otherwise. We conducted all the experiments for 300 s unless specified otherwise.

Scenario #1 (Figure 3a) presents a simple scenario with two separate paths between the sender and receiver. Thus, there are two SFs: SF-1 and SF-2. The path of SF-1 has a bottleneck BW of 10 Mbps, minimum RTT of 14 ms, and packet loss rate of 0.1%, while SF-2 has a bottleneck BW of 5 Mbps, minimum RTT of 24 ms, and loss rate of 0.2%. This scenario challenges the MPTCP-CCAs to utilize the network capacity of both paths simultaneously. In addition, this scenario enables us to observe how well the proposed D-OLIA can estimate the network load and use the available bandwidth in the absence of other traffic.

Scenario #2 (Figure 3b) describes the same network topology as presented in Scenario #1 with a slight difference in the presence of background traffic for SF-1. The properties of the bottlenecks of both the SFs are the same, i.e., they each have a bottleneck BW of 10 Mbps, minimum RTT of 14 ms, and loss rate of 0.1%. This scenario challenges the MPTCP-CCAs to fairly share the SF-1 with single-path TCP traffic while utilizing the SF-2 to its total capacity.

Scenario #3 (Figure 3c) offers a more complex scenario with four SFs, i.e., SF-1 to SF-4. SF-1 and SF-2 share a common bottleneck, while SF-3 and SF-4 share another common bottleneck. The bottlenecks have the same properties, i.e., a bottleneck BW of 10 Mbps, delay of 5 ms, and loss rate of 0.1%. Thus, all the SFs have an equal minimum RTT of 18 ms. Furthermore, bottleneck traffic is present at the common bottleneck of SF-1 and SF-2; as a result, they should occupy a bandwidth reasonably similar to the BW occupied by the background traffic.

### 5.2. Performance Evaluation in Terms of CWND, RTT, and Throughput

In this section, we will evaluate the performance of the considered MPTCP-CCAs in terms of CWND, RTT, and throughput for the considered scenarios.

**Figure 4:**
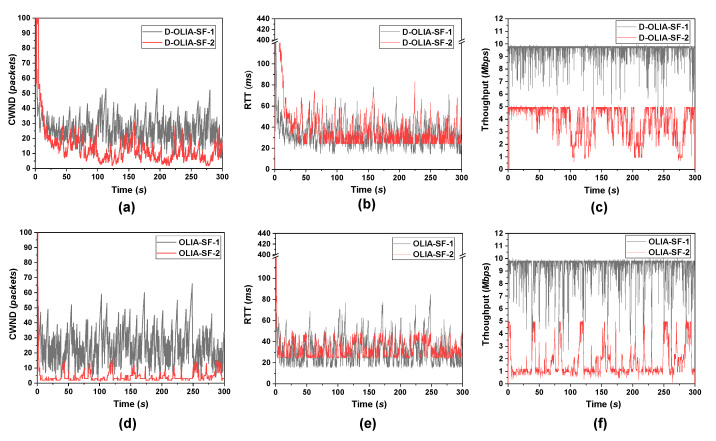
Performance analysis of the considered MPTCP-CCAs for Scenario #1 in terms of CWND, RTT, and throughput for (**a**–**c**) D-OLIA and (**d**–**f**) OLIA.

Figure 4 shows the CWND, RTT, and throughput of D-OLIA and OLIA for Scenario #1. As D-OLIA is a modified version of OLIA, we only showed the performance of D-OLIA and OLIA in this scenario to closely observe the performance improvements provided by D-OLIA. As we can observe in Figure 4a–c, for both the SFs, D-OLIA keeps the RTT low while improving the throughput. As D-OLIA implements network awareness and adaptability to the changing network conditions, whenever the RTT increase is high, D-OLIA rapidly decreases the CWND by halving it, thus releasing the network load quickly. However, when the RTT increase is not high and packet loss occurs, D-OLIA slightly decreases the CWND to keep utilizing the full network capacity. This phenomenon causes D-OLIA to have a higher CWND than OLIA, which enables D-OLIA to have better throughput while ensuring the same or even lower RTT.

**Figure 5:**
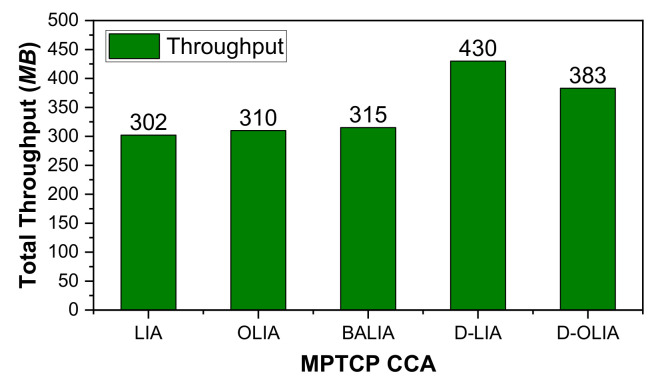
Performance analysis of the considered MPTCP-CCAs for Scenario #1 in terms of total throughput.

Figure 5 shows the total throughput obtained by all the considered MPTCP-CCAs. We can observe that D-OLIA outperforms all the considered MPTCP-CCAs except D-LIA. As mentioned earlier, D-LIA improves the network throughput at the expense of high packet losses because of its lack of awareness about the network condition. D-LIA also results in increased RTTs because of long queueing delays. D-OLIA provides a balance between throughput, high packet losses, and long RTTs by reacting intelligently to packet losses by actively considering the network conditions. The following section will further clarify the performance improvements of D-OLIA with respect to D-LIA.

**Figure 6:**
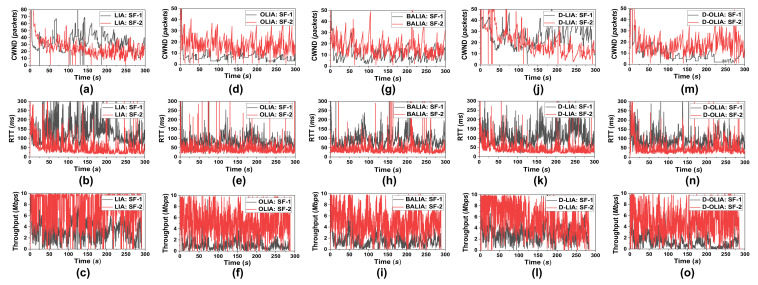
Performance analysis of the considered MPTCP-CCAs for Scenario #2 in terms of CWND, RTT, and throughput for (**a**–**c**) LIA, (**d**–**f**) OLIA, (**g**–**i**) BALIA, (**j**–**l**) D-LIA, and (**m**–**o**) D-OLIA.

**Figure 7:**
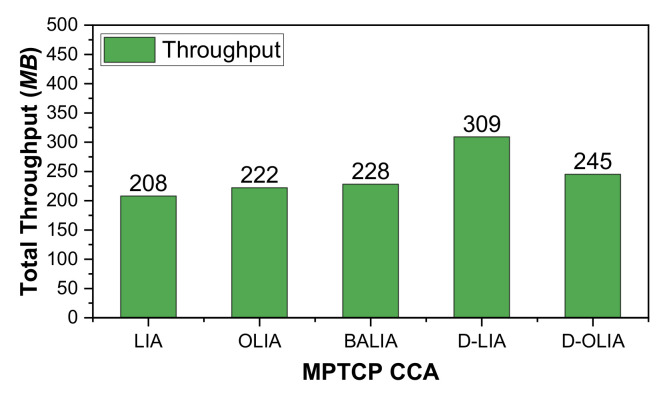
Performance analysis of the considered MPTCP-CCAs for Scenario #2 in terms of total throughput.

Figure 6 shows the CWND, RTT, and throughput for the considered MPTCP-CCAs for Scenario #2. Figure 7 shows the total throughput obtained during the emulation time for the considered MPTCP-CCAs. It is clear that D-OLIA again achieves a better throughput compared to LIA, OLIA, and BALIA while maintaining the low RTT. Here, once again, D-LIA obtains the highest throughput but at the expense of long RTT. This high throughput yield of D-LIA ultimately leads to a significant increase in packet losses, which we will describe in the next section.

**Figure 8:**
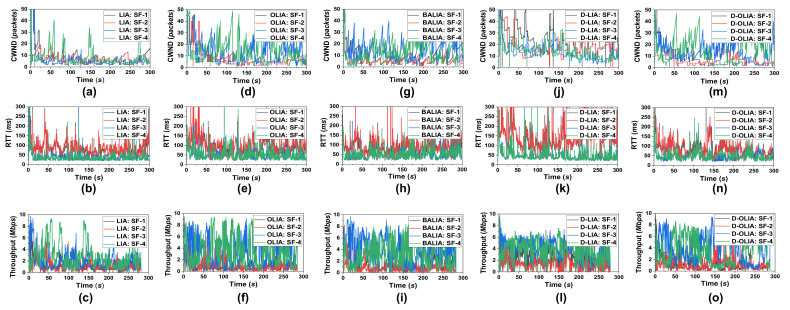
Performance of the considered MPTCP-CCAs for Scenario #3 in terms of CWND, RTT, and throughput for (**a**–**c**) LIA, (**d**–**f**) OLIA, (**g**–**i**) BALIA, (**j**–**l**) D-LIA, and (**m**–**o**) D-OLIA.

**Figure 9:**
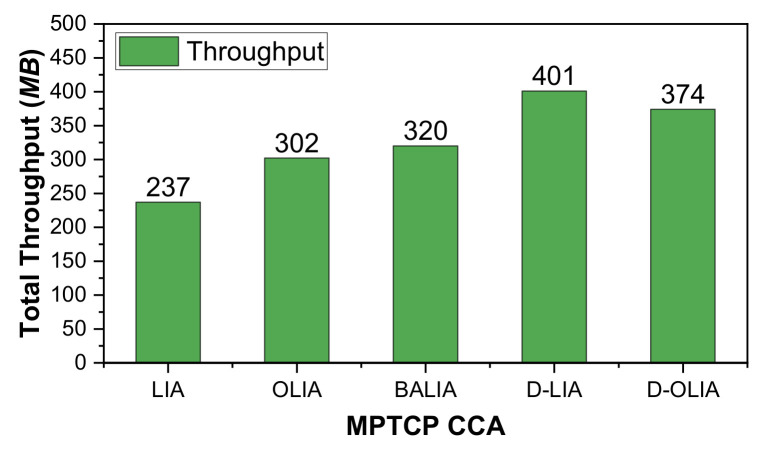
Performance of the considered MPTCP-CCAs for Scenario #3 in terms of total throughput.

Figure 8 shows the performance of the considered MPTCP-CCAs in terms of CWND, RTT, and throughput for Scenario #3. Figure 9 presents the total throughput obtained by the CCAs during the entire emulation time. As we can observe, D-OLIA achieves higher throughput than LIA, OLIA, and BALIA. D-LIA achieves the highest throughput but at the cost of long RTTs, as we can see from Figure 8k. Therefore, D-OLIA is the best performer because it ensures moderately higher throughput than LIA, OLIA, and BALIA, and shorter RTT than D-LIA. Furthermore, the relatively small increase in RTT by D-OLIA than LIA, OLIA, and BALIA seems insignificant considering the increased throughput.

**Figure 10:**
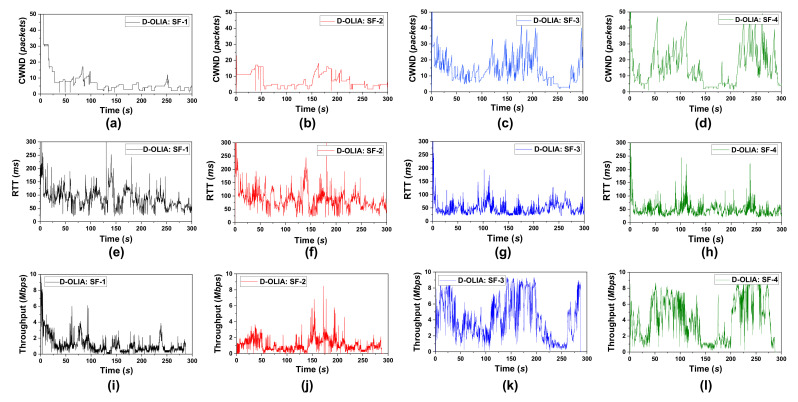
Performance of D-OLIA’s four SFs for Scenario #3 in terms of (**a**–**d**) CWND, (**e**–**h**) RTT, and (**i**–**l**) throughput.

Furthermore, Figure 10 shows the CWND, RTT, and throughput of individual SFs of D-OLIA for Scenario #3. Through close observation, we can see that whenever the RTT becomes significantly prolonged, D-OLIA halves its CWND. When the RTT is moderate, D-LIA slightly decreases the CWND to facilitate high network utilization. For example, considering SF-2, the RTT starts increasing after 133 s in Figure 10f. D-OLIA waits for packet loss events and continuously measures the network condition. At 138 s, during a loss event, D-OLIA decides that the network is congested and halves its congestion window to release the queue, continuing the observation in Figure 10b. Then, it again halves the CWND at 142 s to further free up the bottleneck queue considering the congested network conditions. As the RTT becomes lower, D-OLIA considers the network less-congested and starts the dynamic CWND decrease process in the event of packet losses. In addition, in the simple experiment of OLIA in Section 3, we observed that the later flows fail to achieve equal BW shares in competition with the earlier flows. However, D-OLIA also overcomes this problem by following the hybrid approach. In this experiment, following MPTCP’s connection mechanism, SF-1 is started first, then SF-2 to SF-4 gradually commence one after another. SF-1 and SF-2 share a common bottleneck, while SF-3 and SF-4 share the other common bottleneck. By observing the CWND and throughput curve, it is evident that all the SFs are receiving equal shares following the properties of their bottleneck links.

### 5.3. Performance Evaluation in Terms of Aggregate Benefit, Packet Retransmissions, and Fairness

For an in-depth investigation of the overall performance of D-OLIA in comparison with the considered MPTCP-CCAs, we evaluate their performance in terms of aggregate benefit, the number of total packet retransmissions, and the fairness index.

According to Paasch et al., “Aggregate Benefit (Agr_Bf)” is defined as a parameter that can better apprehend the network utilization by the MPTCP SFs [44]. Agr_Bf is calculated by the following equation:

(8) $$Agr\_Bf=\left\{\begin{array}{ll} \frac{G-B_{\max}}{\sum_{x=1}^{y}B_{x}-B_{\max}} & \;,\;\mathrm{if}\;G\geq B_{\max}\\ \frac{G-B_{\max}}{B_{\max}} & \;,\;\mathrm{if}\;G<B_{\max} \end{array}\right.,$$

where G, $B_{\max}$, $B_{x}$, and *y* correspond to the total goodput of the MPTCP SFs, the maximum available BW among all the SFs, the actual available BW for $SF_{x}$ going through path *x*, and the total number of SFs, respectively. The value of Agr_Bf ranges from −1 to 1; the higher the value, the better the network utilization.

We also calculate the total packet retransmissions by the considered MPTCP-CCAs during the entire emulation time for the three scenarios.

Finally, to analyze how fairly the MPTCP SFs behave with each other, we calculate Jain’s Fairness Index using the following equation [45,46]:

(9) $$\mathrm{Jain}’s\;\mathrm{Fairness}\;\mathrm{Index}=\frac{{\left|\sum_{m=1}^{n}I_{m}\right|}^{2}}{n\sum_{m=1}^{n}I_{m}^{2}},$$

where $I_{m}$ and *n* are the available BW from the total BW of a link to an SF and the total number of SFs going through the link, respectively. The value ranges from 0 to 1. The closer the value to 1, the fairer the SFs are to each other. We calculate Jain’s Fairness Index for the two bottlenecks of Scenario #3.

**Figure 11:**
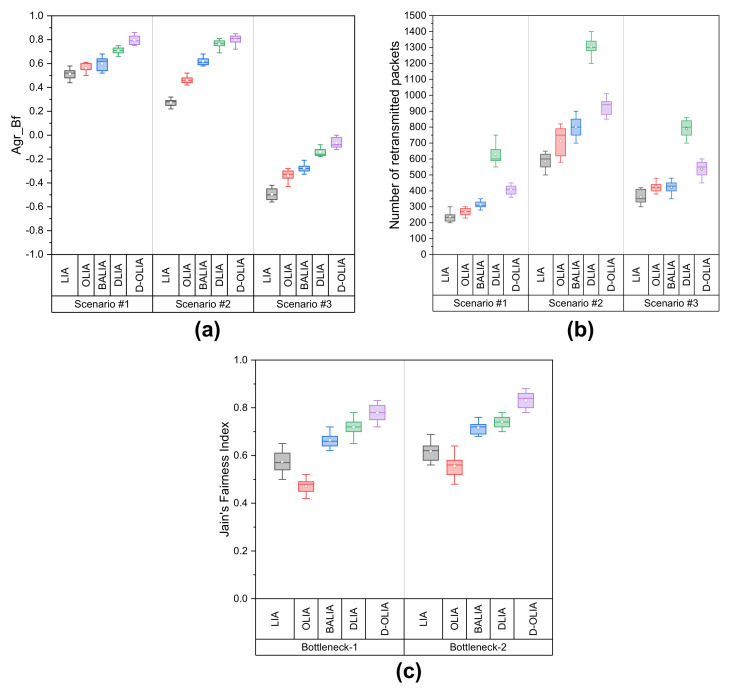
Performance of the considered MPTCP-CCAs in terms of (**a**) Agr_Bf, (**b**) the number of retransmitted packets, and (**c**) fairness among the MPTCP flows in Scenario #3.

Figure 11a–c shows the performance comparison of the considered MPTCP-CCAs in terms of Agr_Bf, number of retransmissions, and Jain’s Fairness Index, respectively. Note that Figure 11 shows the mean with standard deviation and median, at 25% and 75% percentiles, as well as the degree of dispersion.

As observed in Figure 11a, in all the scenarios, D-OLIA performs the best among all the considered MPTCP-CCAs. D-LIA performs slightly lower than D-OLIA in all the scenarios. Although D-LIA obtained the highest throughput, it’s Agr_Bf is lower than D-LIA because of the high packet retransmissions. The calculation of Agr_Bf considers the goodput rather than the throughput. Thus, Agr_Bf reveals the actual performance, excluding the packet retransmission. From the results of Agr_Bf, it also becomes clear that D-LIA obtains a comparatively lower goodput than D-OLIA in all the scenarios.

Furthermore, by observing the number of packet retransmissions in Figure 11b, it becomes clear that D-LIA yields the highest number of packet retransmissions. Although D-OLIA is in the second position, its number of retransmissions is significantly low compared to D-LIA in all three scenarios due to its intelligent network condition estimation and response technique. Compared to LIA, OLIA, and BALIA, D-OLIA yields a slight increase in retransmission but this small increase becomes insignificant compared with the result of Agr_Bf. Thus, in terms of network utilization, D-OLIA performs the best in the scenarios mentioned above. 

Finally, from Figure 11c, considering how fairly the SFs behave amongst themselves, D-OLIA shows the best performance among all the considered MPTCP-CCAs for both the bottlenecks of Scenario #3. We believe that the awareness about the network conditions enables the SFs to allocate a fair share of BW for each other.

### 5.4. Performance Evaluation in a Scalable Network Scenario

In this section, we plan to observe how D-OLIA performs in a scalable network scenario. To emulate a scalable network, we slightly modified Scenario #3 of Figure 3c. Rather than starting only one single-path TCP flow between the single-path sender and receiver, we continued adding new single-path TCP flows after every 20-s interval for 100 s. Then we reduced the number of single-path TCP flows after every 20-s interval. The experiment ended at 180 s. Thus, during the 0–20, 20–40, 40–60, 60–80, 80–100, 100–120, 120–140, 140–160, and 160–180-s periods, there are 1, 2, 3, 4, 5, 4, 3, 2, and 1 single-path TCP flows between the single-path sender and receiver, respectively. Following MPTCP design goals, SF-1 and SF-2 combined should take a BW of around 5 Mbps, 3.3 Mbps, 2.5 Mbps, 2 Mbps, 1.67 Mbps, 2 Mbps, 2.5 Mbps, 3.3 Mbps, and 5 Mbps. At the same time, SF-3 and SF-4 should try to utilize the full capacity of Bottleneck 2. Table 1 summarizes the average throughput obtained by the single-path TCP flows, the combined throughput of SF-1 and SF2, and the total throughput of MPTCP SFs during the 20-s periods. We performed several tests and showed the average results here. Table 1 reveals that D-OLIA successfully allocates a fair share of BW for the single-path TCP flows while sharing a common bottleneck. At the same time, D-OLIA tries to ensure better throughput than the single-path flows by utilizing all the available paths. Therefore, it becomes evident that D-OLIA can successfully adapt to a scalable network and maintain good performance.

## 6. Conclusions

In this work, we attempted to mitigate the drawbacks of the existing MPTCP-CCAs to ensure a fast and reliable Internet. We proposed D-OLIA, a modified version of OLIA, that dynamically decides the CWND decrease factor based on the estimated network condition by analyzing the RTT measurements.

D-OLIA is easily implementable inside the current Linux kernel. We conducted extensive emulation experiments with the Mininet emulator considering various scenarios. D-OLIA could significantly improve OLIA and best utilize the underlying network among the considered MPTCP-CCAs by ensuring high throughput, low delay, and packet retransmissions, as well as ensure fairness among the MPTCP SFs. During the emulation experiments, D-OLIA improved throughput and fairness by 20% and 33%, and decreased RTT by 12% more than OLIA. At the same time, D-OLIA decreased the packet retransmissions by 23% more than D-LIA.

In future work, we plan to further extend the idea for improving the other existing MPTCP-CCAs and compare their impact on ensuring a fast and reliable Internet. 

## Figures and Tables

**Table 1:** In a scalable network scenario, the performance of D-OLIA in comparison with single-path TCP flows in terms of throughput.

	Average throughput of Single-Path TCP Flows (Mbps)	Combined throughput of SF-1 and SF-2 (Mbps)	Total throughput Obtained by all SFs (Mbps)
0–20 s	4.93	4.88	13.33
20–40 s	3.22	3.15	11.91
40–60 s	2.48	2.21	10.55
60–80 s	2.1	1.71	9.61
80–100 s	1.88	1.33	8.28
100–120 s	2.19	1.56	9.22
120–140 s	2.55	2.14	10.37
140–160 s	3.27	3.08	11.44
160–180 s	4.98	4.72	13.08

## Data Availability

Not applicable.
